# Use of Random Domain Intercept Technology to Track COVID-19 Vaccination Rates in Real Time Across the United States: Survey Study

**DOI:** 10.2196/37920

**Published:** 2022-07-01

**Authors:** Rikki H Sargent, Shaelyn Laurie, Leo F Weakland, James V Lavery, Daniel A Salmon, Walter A Orenstein, Robert F Breiman

**Affiliations:** 1 RIWI Corp Toronto, ON Canada; 2 Center for Global Health Innovation Atlanta, GA United States; 3 Hubert Department of Global Health Rollins School of Public Health Emory University Atlanta, GA United States; 4 Center for Ethics Emory University Atlanta, GA United States; 5 Institute for Vaccine Safety Johns Hopkins Bloomberg School of Public Health Baltimore, MD United States; 6 Department of International Health Johns Hopkins Bloomberg School of Public Health Baltimore, MD United States; 7 Department of Health, Behavior and Society Johns Hopkins Bloomberg School of Public Health Baltimore, MD United States; 8 School of Medicine Emory University Atlanta, GA United States

**Keywords:** COVID-19, vaccination rates, Random Domain Intercept Technology, health technology, vaccination, vaccine tracker, web-based survey, epidemiology, health data, digital tool, online intercept sampling, health service

## Abstract

**Background:**

Accurate and timely COVID-19 vaccination coverage data are vital for informing targeted, effective messaging and outreach and identifying barriers to equitable health service access. However, gathering vaccination rate data is challenging, and efforts often result in information that is either limited in scope (eg, limited to administrative data) or delayed (impeding the ability to rapidly respond). The evaluation of innovative technologies and approaches that can assist in addressing these limitations globally are needed.

**Objective:**

The objective of this survey study was to assess the validity of Random Domain Intercept Technology (RDIT; RIWI Corp) for tracking self-reported vaccination rates in real time at the US national and state levels. RDIT—a form of online intercept sampling—has the potential to address the limitations of current vaccination tracking systems by allowing for the measurement of additional data (eg, attitudinal data) and real-time, rapid data collection anywhere there is web access.

**Methods:**

We used RDIT from June 30 to July 26, 2021, to reach a broad sample of US adult (aged ≥18 years) web users and asked questions related to COVID-19 vaccination. Self-reported vaccination status was used as the focus of this validation exercise. National- and state-level RDIT-based vaccination rates were compared to Centers for Disease Control and Prevention (CDC)–reported national and state vaccination rates. Johns Hopkins University’s and Emory University’s institutional review boards designated this project as public health practice to inform message development (not human subjects research).

**Results:**

By using RDIT, 63,853 adult web users reported their vaccination status (6.2% of the entire 1,026,850 American web-using population that was exposed to the survey). At the national level, the RDIT-based estimate of adult COVID-19 vaccine coverage was slightly higher (44,524/63,853, 69.7%; 95% CI 69.4%-70.1%) than the CDC-reported estimate (67.9%) on July 15, 2021 (ie, midway through data collection; *t*_63,852_=10.06; *P*<.001). The RDIT-based and CDC-reported state-level estimates were strongly and positively correlated (*r*=0.90; *P*<.001). RDIT-based estimates were within 5 percentage points of the CDC’s estimates for 29 states.

**Conclusions:**

This broad-reaching, real-time data stream may provide unique advantages for tracking the use of a range of vaccines and for the timely evaluation of vaccination interventions. Moreover, RDIT could be harnessed to rapidly assess demographic, attitudinal, and behavioral constructs that are not available in administrative data, which could allow for deeper insights into the real-time predictors of vaccine uptake–enabling targeted and timely interventions.

## Introduction

Accurate and timely COVID-19 vaccination coverage data are vital for informing targeted, effective messaging and outreach and identifying barriers to equitable health service access. The tracking of vaccination rates is needed in conjunction with effective COVID-19 case surveillance and death monitoring, as well as research on predictors of vaccination uptake and vaccine hesitancy, to optimize vaccine coverage in persons at disproportionate risk of COVID-19 and severe outcomes [1-5]. However, gathering such vaccination rate data is challenging, even in high-income countries such as the United States. Bill Gates recently expressed the importance of investment in global pandemic preparedness [6], which should involve assessing and implementing surveillance systems that can be utilized globally.

In the United States, the Centers for Disease Control and Prevention (CDC) rely on multiple external sources for their vaccination tracker [7]. Although considered the gold standard, this approach is labor intensive and limited to the collection of administrative data. In other words, certain demographic, attitudinal, and behavioral constructs are not available. Another approach involves national polling by using traditional recruitment methods (eg, panels and random digit dialing), in which respondents self-report their vaccination status (eg, Kaiser Family Foundation surveys and the Census Household Pulse) [8,9]. These polls have the benefit of collecting extended information, which could allow for deeper insights into the factors that predict vaccine uptake. Although immensely valuable for assessing knowledge, attitudes, and practices, these polls often do not allow for large-scale, rapid data collection, nor do they provide real-time granular results (with data available daily on a variety of population subcategories). Further, neither of these approaches are easily expandable to other countries with differing monitoring systems and access.

To identify targetable risk factors, it is critical to combine the benefits of traditional survey research with the advantages of rapid and continuous data collection by using a method that is scalable and can be incorporated into a global surveillance system. As such, we assessed the validity of Random Domain Intercept Technology (RDIT; RIWI Corp)—a form of online intercept sampling [10]—for tracking self-reported vaccination rates in real-time, with broad reach, and at the national and state levels across the United States.

## Methods

### Procedure

We used RDIT from June 30 to July 26, 2021, to reach a broad sample of US web users. RDIT can be administered anywhere in the world where there is web access. When web users click on a registered but commercially inactive web link or type in a web address for a site that is dormant, they have a random chance of that link being temporarily managed by the company that owns and administers RDIT—RIWI Corp. In this situation, instead of coming across a “this page does not exist” notification, a survey is delivered. Web users then decide whether to anonymously participate and may exit the survey at any time. No incentives are provided for participation.

Upon encountering the landing page, web users chose their preferred language (English or Spanish), and were told that the survey was anonymous and about COVID-19. Web users who chose to participate first reported their age and gender (those aged under 18 years were immediately exited from the landing page). They then reported their vaccination status (the focus of the validation exercise). Following this, those who were vaccinated were administered questions about the vaccine they received, and those who were unvaccinated were given a series of questions about COVID-19 vaccines (intentions, incentives, barriers, etc). All respondents were asked if they had heard recent accounts of COVID-19 vaccination side effects and were asked to provide information on where they receive their news and entertainment. Except for age, gender, and race and ethnicity, the remaining demographic questions were asked at the conclusion of the survey (education, urban or rural living location, political affiliation, and annual household income).

### Ethical Considerations

This project was approved as public health practice to inform message development (not human subjects research) by the institutional review boards of Johns Hopkins University and Emory University.

### Measures

With regard to COVID-19 vaccination status, respondents were asked “Have you personally received the COVID-19 vaccine?” They were given the following four response options: “Yes, a single-dose vaccine (J&J)”; “Yes, the first of two doses (Moderna or Pfizer)”; “Yes, both doses of a two-dose vaccine (Moderna or Pfizer)”; and “I have not received a vaccine.” RDIT captures nonpersonally identifiable state location information from a respondent’s IP address, which is instantaneously translated into a unique identifier. For this validation analysis, we compared self-reported adult vaccination rates from RDIT (having received at least 1 dose) to those that were provided in the publicly available CDC *COVID-19 Vaccinations in the United States, Jurisdiction* data set on July 15, 2021 (midway through RDIT data collection) [11].

### Statistical Analysis

RDIT-based vaccination rate estimates (and associated logit transformed 95% CIs) were computed for the full national sample (N=63,853) and by state among those with available state information (n=57,986). A series of correlational analyses and 1-sample *t* tests (2-tailed) were used to compare RDIT-based estimates to CDC-reported rates.

## Results

### Respondent Description

By using RDIT, 1,026,850 US web users were exposed to the survey. Of those, 63,853 (6.2%) were aged ≥18 years and reported their vaccination status. Respondents were distributed throughout the sampling window from June 30 to July 26, 2021, with an average of 2365 (SD 1078; range 566-4266; skew=0.04) respondents per day. With regard to gender, 42.4% (27,060/63,853) of respondents were women, 51.6% (32,939/63,853) were men, and 6% (3854/63,853) indicated “other” gender. The median age was 39 (range 18 to ≥85) years. Respondents with available state information were regionally representative (ie, the RDIT-based state sample size strongly and positively correlated with the census-based state population size [12]; *r*=0.98, 95% CI 0.96-0.99; *P*<.001).

Of the 46,955 respondents who reported their race and ethnicity (46,955/63,853, 73.5% of those who reported their vaccination status), 23,505 (50.1%) identified as White; 5702 (12.1%) identified as African American or Black; 5094 (10.8%) identified as Hispanic or Latinx; 3046 (6.5%) identified as Asian; 1367 (2.9%) identified as Native American, American Indian, or Alaskan Native; 4954 (10.6%) identified as multiracial; and 3287 (7%) identified as a nonspecified racial and ethnic group. Of the 14,801 respondents who reported their annual household income (14,801/63,853, 23.2% of those who reported their vaccination status), 6700 (45.3%) reported an income of US $50,000 or less, 4914 (33.2%) reported an income of US $50,001 to US $125,000, and 3187 (21.5%) reported an income of US $125,001 or more. The race and ethnicity and economic distributions observed among the respondents are comparable to the most recent (2020) estimates derived from the US Census Current Population Survey [13,14].

### National Comparison

The national CDC-estimated vaccine coverage on July 15, 2021, was 67.9%. The RDIT-based estimate among the full sample (N=63,853) from June 30 to July 26, 2021, was slightly higher (44,524/63,853, 69.7%; 95% CI 69.4%-70.1%; *t*_63,852_=10.06; *P*<.001).

### State Comparison

The RDIT-based and CDC-reported state-level estimates were strongly and positively correlated (*r*=0.90, 95% CI 0.83-0.94; *P*<.001). RDIT-based estimates were higher than the CDC-reported estimates by a mean of 3% (SD 4.5%, 95% CI 1.7%-4.2%; *t*_50_=4.71; *P*<.001). RDIT-based estimates were higher than the CDC’s estimates for 37 states and were within 2 and 5 percentage points for 12 and 29 states, respectively. When considering the absolute value of the estimate discrepancies, states with more RDIT respondents were associated with smaller discrepancies (*r*=−0.43, 95% CI −0.64 to −0.18; *P*=.001). We observed the largest discrepancy for Alaska (percent difference: 14.5%), which was likely due in part to the small sample size (n=85). The states with RDIT estimates within 1 percentage point of the relative CDC estimate were California; Connecticut; Washington, District of Columbia; Florida; Maryland; Maine; New York; Texas; and Utah (Table 1).

**Table 1:** State-level Random Domain Intercept Technology (RDIT)–based and Centers for Disease Control and Prevention (CDC)–reported adult vaccination rates (at least 1 dose received).

State	Total RDIT respondents, N	RDIT vaccinated respondents, n	RDIT-based vaccination rate, % (95% CI)	CDC-reported vaccination rate, %	Difference^a^, %
AK	85	66	77.6 (67.5-85.3)	63.1	14.5
AL	742	425	57.3 (53.7-60.8)	51.2	6.1
AR	407	248	60.9 (56.1-65.6)	54.2	6.7
AZ	1422	927	65.2 (62.7-67.6)	63.1	2.1
CA	6483	4930	76 (75-77.1)	76.2	−0.2
CO	1026	761	74.2 (71.4-76.8)	70.7	3.5
CT	556	441	79.3 (75.7-82.5)	80.3	−1
DC	270	201	74.4 (68.9-79.3)	73.8	0.6
DE	177	136	76.8 (70-82.5)	71.1	5.7
FL	5275	3518	66.7 (65.4-68)	66.1	0.6
GA	2054	1288	62.7 (60.6-64.8)	55.4	7.3
HI	264	202	76.5 (71-81.2)	84.1	−7.6
IA	420	297	70.7 (66.2-74.9)	64.5	6.2
ID	239	155	64.9 (58.6-70.7)	53.6	11.3
IL	2084	1483	71.2 (69.2-73.1)	73	−1.8
IN	1035	632	61.1 (58.1-64)	57.2	3.9
KS	442	298	67.4 (62.9-71.6)	63	4.4
KY	621	373	60.1 (56.2-63.9)	62.3	−2.2
LA	803	453	56.4 (53-59.8)	50	6.4
MA	1481	1189	80.3 (78.2-82.2)	83.2	−2.9
MD	1186	909	76.6 (74.1-79)	75.9	0.7
ME	182	144	79.1 (72.6-84.4)	78.4	0.7
MI	1658	1110	66.9 (64.6-69.2)	63.2	3.7
MN	903	665	73.6 (70.7-76.4)	70.7	2.9
MO	918	571	62.2 (59-65.3)	57	5.2
MS	355	197	55.5 (50.3-60.6)	47.7	7.8
MT	112	72	64.3 (55-72.6)	59.1	5.2
NC	1927	1253	65 (62.9-67.1)	60.4	4.6
ND	76	48	63.2 (51.7-73.3)	56.1	7.1
NE	338	219	64.8 (59.5-69.7)	65.9	−1.1
NH	181	141	77.9 (71.3-83.4)	74.3	3.6
NJ	1936	1454	75.1 (73.1-77)	77.4	−2.3
NM	346	251	72.5 (67.6-77)	77.7	−5.2
NV	717	492	68.6 (65.1-71.9)	63.2	5.4
NY	4350	3225	74.1 (72.8-75.4)	73.6	0.5
OH	1622	1069	65.9 (63.6-68.2)	59.8	6.1
OK	630	354	56.2 (52.3-60)	58	−1.8
OR	718	541	75.3 (72.1-78.4)	70.7	4.6
PA	1964	1421	72.4 (70.3-74.3)	76.7	−4.3
RI	313	228	72.8 (67.6-77.5)	76.9	−4.1
SC	725	470	64.8 (61.3-68.2)	55.4	9.4
SD	109	73	67 (57.6-75.2)	64.9	2.1
TN	1206	728	60.4 (57.6-63.1)	53.5	6.9
TX	4837	3051	63.1 (61.7-64.4)	62.5	0.6
UT	529	349	66 (61.8-69.9)	66.3	−0.3
VA	1855	1426	76.9 (74.9-78.7)	72.1	4.8
VT	67	61	91 (81.4-95.9)	85.9	5.1
WA	1292	946	73.2 (70.7-75.6)	75.5	−2.3
WI	731	523	71.5 (68.2-74.7)	66.2	5.3
WV	231	146	63.2 (56.8-69.2)	54.5	8.7
WY	86	50	58.1 (47.4-68.1)	50.9	7.2

^a^Difference = RDIT-based vaccination rate − CDC-reported vaccination rate.

## Discussion

### Principal Findings

RDIT provided similar estimates to those provided via the CDC method (the current standard) for vaccination rates at the national and state levels; however, estimates from RDIT are accessible daily at variable magnitudes and have region-targeting capabilities. Although the July 2021 RDIT-based national estimate was higher than the CDC estimate by 2%, it was more comparable to the CDC estimate than those derived from the Census Household Pulse and Delphi-Facebook surveys, which overestimated vaccination coverage by 17% and 14% in May 2021, respectively [15]. At the state level, RDIT-estimated rates strongly correlated with CDC reports, were slightly higher on average, and were within a 5% margin for 57% (29/51) of the states. These findings provide early evidence of the validity of RDIT as a complementary surveillance mechanism for tracking COVID-19 vaccination coverage across the United States.

### Limitations

There are limitations to RDIT that should be considered. First, RDIT only reaches the web-using population; nonetheless, RDIT reaches a diverse set of the web-using population, including respondents who are not habitual survey takers, thereby allowing for subgroup analyses as needed. Second, a repeated measures assessment (ie, a follow-up to assess changes in vaccine status per individual) is not possible because RDIT does not collect identifying information. However, population-level changes can be identified. Similarly, without identifying information, opt-in bias is unknown, but one can record and evaluate trends in retention throughout the survey. Additionally, while associated with analytic limitations, the anonymous nature of RDIT is a strength, as participant privacy is prioritized. Although RDIT enables the collection of additional factors that may provide targets for improving vaccine coverage, it is possible for participants to drop out before they provide this information. An analysis of such drop-off can provide further understanding, and researchers can decide whether it is most appropriate to draw insights from all available data or only from respondents who complete the entire question set (in this case, we chose the former). Finally, administrative data, such as the data that inform the CDC vaccination tracker, could be less susceptible to self-reporting bias; however, we found strong correlations between the self-reported vaccination rate estimates and the CDC administrative metrics.

### Conclusion

Access to this broad-reaching data stream is potentially less labor intensive than the alternative approaches that are currently used by the CDC, and RDIT-based estimates demonstrate adequate accuracy when compared to CDC estimates. RDIT’s real-time nature may be a valuable tool for tracking vaccine uptake; pinpointing localities for targeted, timely interventions; and enabling the rapid evaluation of interventions and messaging campaigns globally. Of course, further investigation is needed to assess the accuracy of RDIT for tracking vaccination status globally. Nonetheless, RDIT could be harnessed to rapidly assess demographic, attitudinal, and behavioral constructs that are not available in administrative data, which could allow for deeper insights into the real-time predictors of COVID-19 vaccine uptake. Such data could be translated into effective interventions to strengthen vaccination coverage.

## Abbreviations

CDC: Centers for Disease Control and Prevention
RDIT: Random Domain Intercept Technology
